# A Systematic Review on Cardiometabolic Risks and Perinatal Outcomes among Pregnant Women Living with HIV in the Era of Antiretroviral Therapy

**DOI:** 10.3390/v15071441

**Published:** 2023-06-26

**Authors:** Perpetua Modjadji, Kabelo Mokgalaboni, Engelbert A. Nonterah, Sogolo Lucky Lebelo, Zandile June-Rose Mchiza, Sphiwe Madiba, Andre Pascal Kengne

**Affiliations:** 1 Non-Communicable Diseases Research Unit, South African Medical Research Council, Tygerberg, Cape Town 7505, South Africa; 2 Department of Life and Consumer Sciences, College of Agriculture and Environmental Sciences, University of South Africa, Florida Campus, Johannesburg 1709, South Africa; 3 Navrongo Health Research Centre, Ghana Health Service, Navrongo P.O. Box 114, Ghana; 4 Faculty of Health Sciences, University of Limpopo, Polokwane 0700, South Africa

**Keywords:** HIV, antiretroviral therapy, pregnant women, cardiometabolic risks, perinatal outcomes

## Abstract

Antiretroviral therapy (ART) regimens have been shown to cause metabolic changes in people living with HIV (PLWH), predisposing them to cardiometabolic disease (CVMD). However, such evidence is less established in pregnant women living with HIV (pWLWH) on ART. Pregnancy-induced cardiometabolic risks (CMR) can predispose to unfavourable pregnancy outcomes and further persist in the postpartum period, resolve, and recur in subsequent pregnancies, or emerge as newly diagnosed chronic diseases of ageing. Therefore, this systematic review aimed at synthesizing evidence on CMR and perinatal outcomes among pWLWH in the era of ART. We considered prospective and retrospective cohorts, case-control, cross-sectional, and interventional studies published in English. Specific keywords were used to conduct a thorough literature search on PubMed-Medline and Scopus following the Preferred Reporting Items for Systematic Review and Meta-Analysis guideline. Two investigators independently screened the search outputs and reviewed full texts of potentially eligible articles. Data extraction was conducted by one investigator and verified by the second investigator. Thirty-one relevant studies conducted on 20,904 pWLWH on ART across Africa, Asia, Europe, and America were included. Studies demonstrate inconclusive findings, especially on perinatal outcomes, but significant risks of gestational hypertension and dyslipidemia were reported in pWLWH on ART compared to the control group. Therefore, future studies should focus more on these perinatal outcomes, and their impact on postpartum maternal health and growth trajectories of uninfected infants born from pWLWH who are either on ART or ART-naïve in comparison to infants born of HIV-negative mothers over the life course, especially in HIV-burdened African countries.

## 1. Introduction

HIV/AIDS has become a global phenomenon [1] and one of the world’s serious public health problems since the first cases were reported in 1981 [2,3]. In particular, more than 17 million women are living with HIV globally, and most are of childbearing age [3]. Every year, 1.4 million of these women experience pregnancies, which without any intervention, carry a risk of vertical transmission of 15% to 45% to the infant [4,5]. However, a combination of antiretroviral therapy (cART) for pregnant women living with HIV (pWLWH) has successfully improved maternal health and prevented mother-to-child transmission (pMTCT) and subsequently outweighed the adverse effects [6,7]. According to the World Health Organization (WHO), the dolutegravir (DTG) ART-based regimens, now used in most low-and-middle income countries (LMICs) as of 2019, is reportedly safe as first-line and second-line treatment in pregnant women [8]. However, in May 2018, a potential safety concern was reported in a study from Botswana reporting four cases of neural tube defects on offspring born of women who became pregnant while taking DTG [9]. Based on these preliminary findings, many countries advised pregnant women and women of childbearing potential to take efavirenz (EFV) instead [8]. Interestingly, a follow-up update from the same observational studies reported a low risk of neural tube defects [10], and some researchers have shown that pharmacokinetic changes for DTG in late pregnancy are not clinically relevant, and support the use of dolutegravir 50 mg once daily with food in pregnancy [11].

Prior to WHO recommending DTG as the preferred first and second-line treatment for all populations, including pregnant women and those of childbearing age [8], there was an increase in the variety of ART, and it was unclear which ones were effective [12]. However, previous data suggested that first-line ART regimens [TDF + FTC + non-nucleotide reverse transcriptase inhibitors (NNRTI) EFV] were safe during pregnancy [13]. Therefore, considering the implementation of DTG as the first and second-line regimens, especially in high-burden countries, research on its effect on birth outcomes is important as most countries have now transitioned to DTG [8]. Moreover, pregnant women who start ART prior to the onset of their pregnancy have different baseline biochemical characteristics than women who start ART during pregnancy [12]. Although evidence shows a high prevalence of cardiometabolic risk (CMR) in pWLWH, little is known about the effect of ART on their cardiometabolic health [14,15,16,17]. Some possible mechanisms involved may include complex interactions of HIV, ART, and chronic inflammation [18,19]. 

The concern remains that CMR can persist in the postpartum period, resolve, and recur in subsequent pregnancies [19]. These risks can also emerge as newly diagnosed chronic diseases of ageing and persist over the life course [19]. Noteworthy is that during pregnancy, hypertensive disorders of pregnancy exist on a spectrum from preexisting, chronic hypertension to gestational hypertension, preeclampsia, eclampsia, and the syndrome of hemolysis, elevated liver enzymes, and low platelet count (HELLP) [18]. In contrast, hyperglycemic disorders in pregnancy range from preexisting type 1 or type 2 diabetes mellitus and gestational diabetes mellitus (GDM) to new diabetes mellitus in pregnancy, which is distinguished from GDM by its severity and persistence postpartum [20]. On the other hand, the initiation of ART is often associated with weight gain, which has been referred to as a “return to health” phenomenon among those who are underweight when starting treatment [21]. However, the degree of weight gain varies by ART regimen and has been associated with the development of metabolic syndrome, diabetes, and cardiovascular diseases [22]. 

Furthermore, the potential adverse effects of in utero ART exposure remain elusive, while the association between untreated, advanced HIV disease, and adverse birth outcomes is well documented [23,24,25]. Studies have reported unfavourable perinatal outcomes, including increased spontaneous miscarriages, preterm, stillbirths, increased perinatal mortality, intrauterine growth restriction, low birth weight, and chorioamnionitis [26,27,28,29,30]. Specifically, offspring born at the lowest birth weights who experience rapid postnatal growth are at the greatest risk for later life morbidity and mortality, suggesting that a mismatch between environments in the perinatal period and infancy or early childhood leads to molecular alterations that persist over the life course [31,32]. The aforementioned circumstances might affect offspring negatively [27,33], and further exacerbate a higher risk of suboptimal growth in infancy [23,28,34,35,36], even to school age [23,28]. Infants who are HIV-exposed but uninfected have poorer growth, health, and survival outcomes, as well as increased risk of infectious morbidity and mortality compared to their counterparts [37,38,39,40]. It is acknowledged that the cause of this increased morbidity in HIV-exposed and uninfected infants is multifactorial, but in utero exposure to ART may be a contributing factor. Therefore, the association between maternal exposure and the timing of initiation to ART and adverse outcomes are still in question. This systematic review aimed at synthesizing evidence on CMR and perinatal outcomes among pWLWH in the era of ART.

## 2. Methodology

### 2.1. Study Design

A systematic review methodology was used to assess the evidence from clinical studies that investigated perinatal outcomes and cardiometabolic parameters in pWLWH on ART, and the focus was on hypertension and gestational hypertension, following the guideline of the preferred reporting items for systematic review and meta-analysis (PRISMA) [41].

### 2.2. Eligibility Criteria

The systematic review only focused on evidence from peer-reviewed clinical studies published in English. There were no limitations regarding study designs; prospective, retrospective cohorts, case-control, cross-sectional, observational, and interventional studies were considered. Preclinical studies, reviews, conference abstracts, editor’s notes, commentaries, and unpublished work were not considered. Our population, intervention, comparator and outcome (PICO) criteria were as follows: P, HIV-positive pregnant women; I, ART; C, HIV-negative pregnant women/healthy women; O, cardiometabolic factors and perinatal outcomes.

### 2.3. Literature Search

A thorough literature search was made using electronic databases such as PubMed-Medline and Scopus by two investigators (KM and PM) following the guideline of the Preferred Reporting Items for Systematic Review and Meta-Analysis [41]. Due to the prevalence of HIV in South Africa and globally, we searched for evidence without any limitation in publication dates and regions. Therefore, the evidence was searched from inception until March 2023. We additionally searched for studies through a manual screening of relevant studies and reviews. The search strategy was developed and adapted in both databases with the assistance of the librarian. Medical subject heading (MeSH) terms, synonyms, and Boolean operators used included “HIV”, “HIV-1”, “HIV-2”, “HIV infections”, “AIDS”, “Acquired Immunodeficiency Syndrome”, “pregnant women”, “pregnancy outcomes”, “pregnancy complications”, “pregnancy characteristics”, “pregnancy problems”, “ARV”, “ART”, “Anti-HIV Agents”, “HIV Protease Inhibitors”, “HIV Integrase Inhibitors”, “highly active antiretroviral therapy”, “combination ART”, “combination ARV” “perinatal”, “cardiovascular risk”, “AND”, and “OR”. A detailed search strategy and MeSH terms adapted from PubMed and Scopus are presented in Supplementary Tables S1 and S2. The flow diagram showing the study selection was created with the use of the R package and Shiny App [42].

### 2.4. Study Selection

Investigators (KM and PM) screened all studies identified from databases based on title, abstract, and keywords. This was followed by retrieving full text and thorough screening based on our eligibility criteria. Where required due to disagreement, a third investigator (EAN) was consulted for an independent opinion as to whether the study was relevant based on our eligibility criteria. 

### 2.5. Data Extraction and Synthesis

From all relevant studies, KM and PM independently extracted the following information, author and year of publication, country of publication, study design, population status, and cardiometabolic markers using a standard form created based on our eligibility criteria. Both investigators compared the spreadsheet after completing the extraction and any inconsistencies were resolved based on the opinion of the third independent investigator (EAN).

## 3. Results

### 3.1. Search Strategy, Selection Criteria, and Overall Features of Included Studies

**Figure 1:**
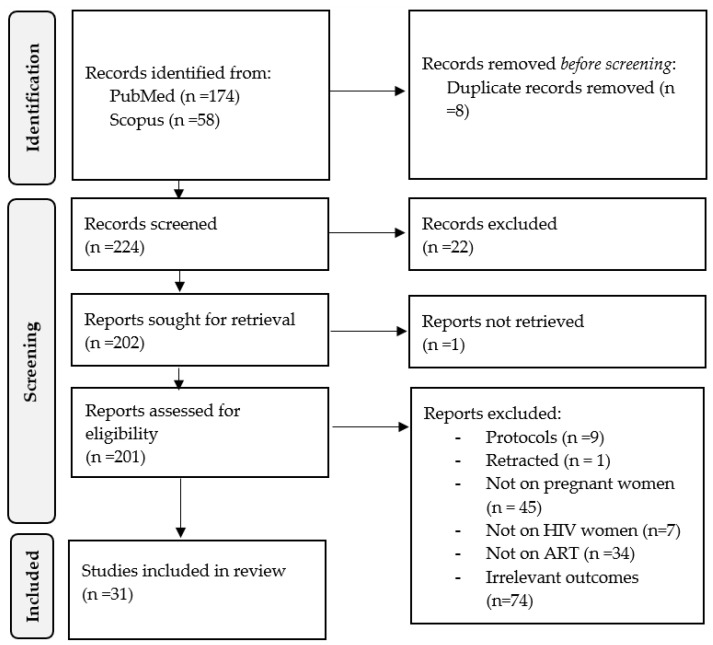
PRISMA flow diagram showing study selection based on eligibility criteria.

Two hundred and thirty-two records were identified from online databases and subjected to screening following eligibility criteria. Eight duplicates identified by the reference manager were removed, and the remaining 224 records were screened by title, abstract, and keywords according to the inclusion criteria. During this stage, 22 studies were excluded as they were deviating from the research in question in terms of title, aims, and keywords. In the next phase, one record was not available, and means were made to contact the corresponding author to request full text without response. Therefore, 201 full texts were retrieved and subjected to screening, from which nine were excluded as they were research protocols, one was retracted from a journal, 45 did not include pregnant HIV women, seven were not on HIV, 34 were not on ART, and 74 did not report any outcome of interest, such as any outcome of perinatal or marker of CMR. Only 31 clinical studies [12,26,33,36,43,44,45,46,47,48,49,50,51,52,53,54,55,56,57,58,59,60,61,62,63,64,65,66,67,68,69] conducted on 20,904 HIV women on ART before or during pregnancy were found relevant based on our eligibility criteria (Figure 1). These clinical studies were published in peer-reviewed journals between 1999 and 2023. The publications were from Southern Africa (South Africa [45,52,69] and Botswana [66], Eastern Africa (Ethiopia [36], Tanzania [69], Kenya [64], Uganda [60,62,69], Mozambique [33,61], Malawi [69], Zambia [52,69], and Zimbabwe [69]), Southern Europe (Spain [46,47]), Northern Europe (United Kingdom [55]) and Western Europe (Netherlands [12,57]), Eastern Asia (China [26,67]), Southern Asia (India [69] and Thailand [49]), Southern America (Brazil [43,50,51,56]) and Northern America (Canada [60] and USA [44,48,53,54,58,59,60,63,65,68]). The characteristics of included studies are presented in Table 1.

### 3.2. Synthesis of Evidence

#### 3.2.1. Antiretroviral Therapy and Neonatal Outcomes

Preterm delivery, preterm birth, low birth weight, small for gestational age, gestational diabetes, preeclampsia, fetal growth restriction, stillbirth, aberrant fetal well-being, and HIV mother-to-child transmission were among the perinatal outcomes evaluated in the included studies. The results generated from this study showed conflicting findings in terms of neonates’ outcomes, especially in infants born from HIV-infected women who were on ART during their pregnancy. For instance, some studies showed no association between HIV/ART and the risk of perinatal outcomes in pWLWH. Madlala et al. [45] reported that maternal obesity was associated with an increased risk of having high birth weight and large size for gestational age infants. The same cohort showed that gestational weight gain was associated with an increased risk of spontaneous preterm delivery and high birth weight infants [45]. At least eight studies showed no significant differences in the prevalence of maternal death, preterm delivery, low birth weight, and neonatal HIV infection [12,27,33,43,47,54,60,65]. Other studies showed a significant increase in the risk of perinatal outcomes, including high birth weight, preterm delivery, mother-to-child infection, and stillbirth [36,51,55,56,57,61,62,63,64,66].

#### 3.2.2. Antiretroviral Therapy and Cardiometabolic Risk

Cardiometabolic risk factors increase the risk of developing cardiovascular diseases, especially in HIV patients on protease inhibitors-based ART, such as ritonavir and lopinavir, and NNRTI-based ART such as efavirenz [70]. Evidence showed that pWLWH had an increased risk of developing hypertension, especially at ≤ or greater than 20 weeks of gestation [44]. Dyslipidemia was also significant in HIV women on ART compared to PMTCT during antenatal care [49].

## 4. Discussion

The current systematic review found inconclusive results in terms of the risks of preterm delivery, low birth weight and SGA, as other studies reported an increased risk in pWLWH and on ART compared with HIV-negative controls or those on placebo and vice versa. Additionally, hypertension and dyslipidemia were observed in pWLWH on ART. Globally, pWLWH using ART may have changes in the foetus’ or the baby’s cardiac development. However, this is worse in African countries, considering the lack of advanced resources and has been classified as low in South Africa. A previous quantitative analysis by Wedi et al. [71] reported an increased risk of preterm birth, low birth weight, SGA, and stillbirth in HIV-pregnant women not on ART. Similarly, Shinar demonstrated an increased risk of preterm birth, low birth weight, and SGA in HIV-pregnant women on ART compared with an uninfected group [72]. 

Although Osmundo et al. reported no risk of perinatal outcomes, they indicated that perinatal-acquired HIV was associated with the risk of preterm birth in the trimester [43]. Similarly, other researchers also reported a significant risk of preterm birth in pWLWH when compared to uninfected pregnant women [26,46,55,59,62]. The results are supported by findings from a longitudinal study in South Africa, which also demonstrated a significant increase in the risk of preterm delivery [45]. Although this was consistent with the findings from other researchers, it seems the findings are attributable to the maternal health states, as these women were obese. Studies have shown an association between maternal obesity and spontaneous preterm labour [73]. Although the exact mechanism is unclear, it is speculated that preterm in pregnant obese women may be mediated by gestational diabetes and hypertensive disorders of pregnancy [74]. Interestingly, a meta-analysis also reported that maternal obesity is associated with large for gestation age and premature birth, whereas maternal underweight was associated with small for gestational age and low birth weight [75]. Moreover, the prevalence of obesity is rapidly increasing in pregnant women [76,77,78]. Altogether, these suggest that approaches that can reduce body weight or control obesity in pWLWH can be a relevant and useful approach to prevent the risk of preterm birth. 

Conflicting results were reported most recently by another group [33,61] that showed no significant differences in preterm delivery and neonatal HIV infection between women with and without advanced HIV diseases. On the other hand, Lallemant et al. [67] reported no significant difference birth weight and preterm delivery, suggesting that the HIV states of the women did not influence the overall health of the infant. ART is generally prescribed to PWLWH to prevent perinatal transmission and has, in the past, shown positive results [79]. Several mechanisms are implicated by which ART reduces perinatal transmission; for instance, ART lowers the maternal antepartum viral load [80].

Additionally, ART prophylaxes are given to infants born from HIV-infected women who were not on ART to reduce the risk of the baby becoming infected with HIV [80]. In the current review, we found contradicting reports by different researchers on the effect of ART on prenatal outcomes. For instance, one report showed that cART in pWLWH did not increase the risk of premature delivery compared to the group on monotherapy or the untreated group [48]. These results were further supported by McDonald et al., although these were compared between efavirenz and lopinavir/ ritonavir-based ART [60]. 

On the other hand, there was a high risk of preterm delivery and other neonatal adversities when pWLWH took ART. For example, PI-based HAART was reported to have a high risk of preterm delivery; however, this was associated with no mortality or hospitalization [63]. A prospective study from Uganda also revealed that pWLWH initiating cART during pregnancy and gaining at least 0.1 kg per week were at high risk of having preterm delivery and low birth weight [62]. These findings again point out the implications of obesity in pregnant women, which subject them to obese-associated complications. Ejigu et al. [36] also reported an increased risk of preterm birth in women who initiated HAART during pregnancy compared with previously used zidovudine as a monotherapy. The same cohorts further revealed that nevirapine-based HAART was associated with a higher risk of preterm births than efavirenz-based HAART. Similar findings were observed from another cohort [57], which reported increased preterm delivery after the first trimester in women on HAART. Consistent results from the prospective cohort also support the prior finding, although this research study was conducted in Brazil. For instance, pWLWH on ART pre-conception reportedly had a high risk of preterm delivery due to low birth weight [56]. Moreover, other researchers also demonstrated a significantly increased risk of low preterm birth in ART-exposed women compared to ART-naïve [51]. The use of cART or participation in the PMTCT was substantially associated with preterm delivery during labour [49]. 

However, Tuomala et al. [48] have found contradicting results as demonstrated by no association between the use of cART and the risk of premature delivery, low birth weight, or stillbirth compared to the untreated group. Consistent findings were reported by other scholars [54]; for example, this group indicated that there were no significant differences by regimen in the individual outcomes of stillbirth, neonatal death, preterm birth, very preterm birth, and SGA when compared to the counterpart group. Li et al. [26] showed that mono or dual therapy and HAART protected stillbirth when most HIV-infected pregnant women started ARV therapy during or after the second trimester. 

Previous clinical evidence has revealed a close association between HIV infection in pregnancy and a significant risk of low-birth-weight infants [81,82]. Despite the controversies about the effect of ART on perinatal outcomes, adequate evidence suggests that the use of ART in pWLWH increases the risk of adverse perinatal outcomes. All these contradicting findings suggested that HIV and ART may independently induce adverse perinatal outcomes. A previous study in Zimbabwe showed that infants with birth weights of <2.5 kg had an increased risk of HIV-positive outcomes than those with birth weights of >2.5 kg [83]. Although the exact interaction mechanism between maternal HIV and low infant birth weight is unclear, one possible cause is HIV-induced placental inflammation, which seems to disrupt placental functions such as maternal-foetal exchange [84]. 

Rollins and colleagues showed risk of adverse pregnancy outcomes such as death of low-birth weight infants, in pWLWH in South Africa. The same results are seen in pWLWH on ART [25], which is still consistent, despite the advancement in ART in developing countries like South Africa. For instance, Madlala et al. [45] reported that pregnant women who are obese also have an increased risk of having high birth weight and large size for gestational age infants. The same study further showed that gestational weight gain was closely associated with the risk of high birth weight infants. HIV-exposed, uninfected infants have an increased risk of morbidity and mortality from perinatal HIV and ART exposure, despite the availability of advanced forms of ART. Therefore, frequent monitoring and reporting are important to protect this susceptible group in our everchanging HIV treatment and prevention approach [85]. 

One of the limitations includes the inconsistent findings on clinical trials exploring the effects of ART on cardiometabolic and perinatal outcomes in pWLWH. Despite a rigorous search for evidence, these aspects have some paucity. Nevertheless, independent investigators were involved in each step of the search, selection, and extraction to avoid the risk of bias that could have arisen in the process. Moreover, the gathered evidence was spread across different countries that showed different findings, thus, excluding the risk of associated publication bias.

## 5. Conclusions

The findings in this systematic review revealed inconclusive evidence, especially on perinatal outcomes, as some studies reported increased risk, while others reported no risk. However, compared to the control group, there were significant risks of gestational hypertension and dyslipidemia in pWLWH on ART. Therefore, prospective future studies should focus more on these perinatal outcomes and their impact on postpartum maternal health and growth trajectories of uninfected infants born from pWLWH who are either on ART or ART-naïve in comparison to infants born of HIV-negative mothers, especially in HIV-burdened African countries. These studies should further consider potential long-term health consequences throughout the life course in the era of the dolutegravir ART regimen; a recent first and second-line treatment in most countries recommended by the World Health Organization.

## Figures and Tables

**Table 1:** Basic characteristics of included studies (n = 31) on HIV pregnant women treated with antiretroviral therapy globally.

Authors, Year of Publication	Country	Study Design	Population States	Cardiometabolic Markers and Perinatal Outcomes Reported	Summary of Findings
(Osmundo et al., 2023) [43]	Brazil	Retrospective cohort	186 pWLWH on ART.	Preterm birth, low birth weight, foetal loss, mother-to-child transmission.	Pregnant women living with HIV (pWLWH) did not increase the risk of adverse perinatal outcomes, and preterm birth [OR = 0.7, 95% CI: (0.3–1.8), *p* = 0.499]. However, in the third trimester, anaemia was associated with preterm birth (*p* = 0.039).
Tymejczyk et al., 2022 [44]	USA	Retrospective cohort	1965 pWLWH with about 2306 live births on the PMTCT program.	BMI, body weight, and blood pressure.	Hypertension was increased at about 20 weeks gestation.
Nhampossa et al., 2022 [33]	Mozambique	Prospective, retrospective cohort	260 pWLWH on ART who attended antenatal care.	BMI, preterm birth, low birth weight, neonates deaths.	There were no significant differences in the prevalence of maternal death, preterm delivery [20 (11.9), *p* = 0.187] compared to [99 (8.4)], low birth weight, and neonatal HIV infection between women with and without advanced HIV diseases.
Madlala et al., 2020 [45]	South Africa	Prospective cohort	249 pWLWH on ART during pregnancy.	Hypertension, preterm, stillbirth, low birth weight.	Maternal obesity was associated with an increased risk of having high birth weight and large size for gestational age infants. In the subset cohort, gestational weight gain was associated with an increased risk of spontaneous preterm delivery [OR = 4.35, 95% CI: (1.55–12.21), *p* = 0.005] and high birth weight infants.
Garća-Otero et al., 2016 [46]	Spain	Prospective cohort	42 pWLWH on cART.	Preterm, low birth weight, diastolic and heart rate.	Cardiac remodelling and dysfunction were observed in foetuses from HIV-infected mothers on cART. Moreover, HIV infected group had significantly increased preterm birth [(6.0 ± 14.3), *p* = 0.002] compared to the uninfected-HIV [1.0 ± 1.2] group.
De la Calle et al., 2015 [47]	Spain	Longitudinal cohort	29 pWLWH on HAART.	Systolic and diastolic velocity.	There were no significant differences in foetal cardiac parameters, especially in those born from HIV-infected pregnant women treated with HAART.
Tuomala et al., 2002 [48]	USA	Clinical trial	2123 pWLWH on ART	Stillbirth, low birth weight, premature delivery.	When compared to the group without ART, or monotherapy, the cART group was not associated with an increased risk of premature delivery [OR = 1.80, 95% CI: (0.94–3.43), *p* > 0.05], low birth weight, or stillbirth in their infants.
Areechokchai et al., 2009 [49]	Thailand	Prospective and retrospective cohorts	246 pWLWH on ART.	Dyslipidaemia, preterm, stillbirth.	Compared to antenatal care clinics, a significant increase in the prevalence of preterm delivery was noted in the groups on cART (19.4%) or initiating PMTCT (19%) during labour without antennal care compared to those on PTMCT during antenatal care (6.9%). Significant dyslipidaemia in ART compared to PMTCT during antenatal care.
Barral et al., 2014 [50]	Brazil	Cohorts	262 pWLWH on ART.	Low-birth weight.	ART showed no effect on the outcome of pregnancy. However, initiation of prenatal care after the first trimester showed an effect on low birth weight and increased risk of prematurity.
Santini-Oliveira et al., 2014 [51]	Brazil	Prospective study	36 pWLWH on ART.	Preterm delivery, low birth weight and birth abnormalities.	Low frequency of preterm delivery in ART-exposed [4 (11.1)] compared to ART-naïve [14 (7.8)].
Nyemba et al., 2022 [52]	South Africa, and Zambia	Prospective cohort	395 pWLWH on ART.	Low birth weight.	Length for age was lower among infants who were HIV-exposed-uninfected.
Baltrusaitis et al., 2022 [53]	USA	Open-label randomised controlled trial	479 pWLWH treated with ART at week 14 of pregnancy.	Low birth weight.	The TDF-ART regimen showed no observed safety concerns for maternal or infant renal function during pregnancy.
Zash et al., 2018 [54]	USA	Observational study	6322 pWLWH (1729 dolutegravir) and 4593 on EFV.	Preterm, stillbirth, neonatal deaths, and low-birth weight.	There were no significant differences by regimen in the individual outcomes of stillbirth, neonatal death, preterm birth [RR = 0.98, 95% CI: (0.87–1.11)], very preterm birth [RR = 1.09, 95% CI: (0.82–1.45)], and small for gestational age (SGA).
Snijdewind et al., 2018 [12]	Netherlands	Retrospective and observational study	2184 pWLWH receiving cART.	Gestational age, low birth weight, and preterm delivery.	Women starting cART before conception had an increased risk of having SGA infants compared to women starting cART after conception. There was no significant difference in perinatal death or birth weight between women on cART pre- and post-conception.
Montgomery, 2015 [55]	UK	Retrospective cohort	27 pWLWH who started ART during pregnancy.	Preterm births, low birth weight, stillbirth.	One neonate was diagnosed with HIV infection. There were 6 preterm births, 9 cases of low birth weight, 11 small-for-gestational-age neonates, and 1 stillbirth.
Machado et al., 2009 [56]	Brazil	Prospective cohort	696 pWLWH, 130 on ART before pregnancy, and 566 on ART after conception.	Preterm and low-birth weight hypertension.	Patients on HAART pre-conception had an increased risk of low birth weight and preterm delivery [OR = 2.22, 95% CI: (1.08–4.54), *p* = 0.009].
Ejigu et al., 2019 [36]	Ethiopia	Retrospective cohort study	1663 pWLWH on ART.	Preterm and low birth weight.	A higher risk of preterm birth among women who initiated HAART before pregnancy [OR = 0.93, 95% CI: (0.78–1.29)] compared with zidovudine monotherapy [OR = 0.35, 95% CI: (0.19–0.64)]. Pregnancies exposed to nevirapine-based HAART also had a greater risk of preterm births [OR = 1.44, 95% CI: (1.06–1.9)] than zidovudine-based HAART [OR = 1.16, 95% CI: (0.83–1.62)] and PI-based HAART [OR = 1.81, 95% CI: (0.78–4.18)].
Boer et al., 2007 [57]	Netherlands	Prospective cohort	98 pWLWH on HAART.	Mother-to-child transmission, preterm delivery, low birth weight, preeclampsia.	When compared to the control group, there was an increased in preterm delivery in pWLWH [15 (10%)] compared to 6 (3%) in HIV-negative. HAART was associated with increased preterm delivery observed after the first trimester [OR = 2.84, *p* = 0.04}.
Li et al., 2020 [26]	China	Prospective cohort	414 of 483 pWLWH on ART.	Stillbirth, preterm birth, low birth weight and small for gestational age.	Stillbirth, preterm birth, low birth weight, and SGA were significantly increased by maternal HIV infection but not neonatal asphyxia or birth abnormalities. Compared to untreated HIV infection, mono/dual therapy and HAART protected stillbirth when most HIV-infected pregnant women started ARV therapy during or after the second trimester.
Aaron et al., 2012 [58]	USA	Prospective cohort	183 pWLWH on ART.	Preterm, low birth weight, infant birth weight	Women taking NNRTI had a lower risk of having an SGA infant than women on PIs.
Silverman et al., 1998 [59]	USA	Multicenter, prospective observational study	39 pWLWH on ART.	Birth weight	There were no significant adverse neonatal outcomes except for the three preterm newborns.
McDonald et al., 2018 [60]	Uganda, Canada, and the USA	Randomised controlled trial	326 pWLWH (160 randomised to the EFV arm and 166 women to the LPV-based ART.	Preterm, low birth weight, stillbirth	There was no significant difference on preterm delivery in EFV [24 (15.0), *p* = 0.46] and LPV/r-based ART [31 (18.7)]. There was no significant difference in both groups on low birth weight and stillbirth.
Gonzalez et al., 2017 [61]	Mozambique	Prospective cohort	561 pWLWH on ART.	Stillbirths, congenital malformations, neonatal deaths, low birth weight, and prematurity	The risk of stillbirths was doubled in HIV-infected women. However, no differences between groups were observed in mean birth weight, prematurity, and maternal and neonatal deaths.
Young et al., 2012 [62]	Uganda	Prospective cohort	166 pWLWH, ART-naïve pregnant women were enrolled between 12- and 28 weeks gestation and treated with a protease inhibitor or non-nucleoside reverse transcriptase inhibitor-based combination regimen.	Preterm, stillbirth, low birth weight	In HIV-infected women initiating cART during pregnancy, inadequate gestational weight gain was observed. Infants whose mothers gained 0.1 kg/week were at increased risk for low birth weight, preterm delivery [OR = 3.46, 95% CI: (1.18–10.15), *p* = 0.024], and composite adverse birth outcomes. cART did not reduce the burden of adverse birth outcomes among HIV-infected women.
Powis et al., 2011 [63]	USA	Retrospective cohort	560 pWLWH randomised to ART between 26 and 34 weeks of pregnancy.	Preterm infant death	PI-based HAART was associated with increased preterm delivery [24 (25%)] compared to triple NRTI-HAART [42 (16.7%)] without infant hospitalisations or mortality.
Drake et al., 2012 [64]	Kenya	Randomised, double-blind trial	148 pWLWH coinfected with HSV given 500 mg valacyclovir or placebo beginning at 34 weeks gestation.	Preterm	Infants born from HIV on ART had increased weight compared to placebo.
Mofenson et al., 1999 [65]	USA	Randomised, controlled trial	480 pWLWH on zidovudine.	Birth weight	There was no perinatal transmission of HIV-1 among the 84 women who had HIV-1 levels.
Shapiro et al., 2010 [66]	Botswana	Randomised controlled trial	560 pWLWH on abacavir, zidovudine, and lamivudine at 26 to 34 weeks of pregnancy.	Premature, low birth weight, congenital abnormalities, infants deaths	Only 8 children were HIV-infected at 24 months. The NRTI-treated arm had a high preterm delivery [42/283 (15)] compared to the observational [16/156 (10)].
Lallemant et al., 2015 [67]	China	Randomised, partially double-blind and placebo-controlled trial	405 pWLWH on zidovudine starting at 28 weeks of pregnancy.	Stillbirth, preterm, low birth weight	There was a significant difference in gestation period without difference in birth weight, preterm delivery [21 (14.8%)] in LPV compared to [17 (12.6%)] in NVP, and low birth weight.
Mulligan et al., 2018 [68]	USA	Non-randomised, open-label, parallel-group prospective trial	29 pWLWH and infants on ART.	Preterm, low birth weight	Twenty-nine infants were HIV-negative. Renal abnormalities were noted on ultrasound in two infants associated with the use of dolutegravir.
Aizire et al., 2020 [69]	India, Malawi, South Africa, Tanzania, Uganda, Zambia, and Zimbabwe	Retrospective case-control study	33 pWLWH treated with ART during pregnancy.	Preterm, stillbirth, and infant death.	TFV-DP concentrations in dried blood spots appeared not associated with severe adverse neonatal outcomes, including preterm [OR = 0.96, 95% CI: (0.28, 3.30)], stillbirth and early infant death.

BMI: body mass index; HIV: human immune deficiency virus; pWLWH: pregnant women living with HIV; ART: antiretroviral therapy; HAART: highly active antiretroviral therapy; cART: combined antiretroviral therapy; PMTCT: prevention of mother to child transmissions; PI: protease inhibitors; SGA: small for gestational age; HSV: herpes simplex virus; TFV-DP: tenofovir diphosphate; EFV: efavirenz; NVP: nevarapin; LPV: lopinavir; NNRTI: non-nucleoside reverse transcriptase inhibitors; OR: odds ratio; RR: relative risk; CI: confidence interval.

## Data Availability

Not applicable as this study involved the use of already published studies.

## Supplementary Materials

The following supporting information can be downloaded at: https://www.mdpi.com/article/10.3390/v15071441/s1, Table S1: Search strategy on PubMed/MEDLINE; Table S2: Search strategy on Scopus.
