# Supplementary material for: A Systematic Review on Cardiometabolic Risks and Perinatal Outcomes among Pregnant Women Living with HIV in the Era of Antiretroviral Therapy
Source: Viruses. 2023 Jun 26;15(7):1441. doi: 10.3390/v15071441 (PMC10385451; doi:10.3390/v15071441)
Supplement: Supplementary file 1 [file viruses-15-01441-s001.zip › viruses-2430317-supplementary.pdf]

viruses

Review

A Systematic Review on Cardiometabolic Risks and Perinatal Outcomes among Pregnant Women Living with HIV in the Era of Antiretroviral Therapy

Perpetua Modjadji [1,*], Kabelo Mokgalaboni [2], Engelbert Nonterah [3], Sogolo Lucky Lebelo [2], Zandile June-Rose Mchiza [1,] Sphiwe Madiba [4] and Andre Pascal Kengne [1]

[1] Non-Communicable Diseases Research Unit, South African Medical Research Council, Tygerberg, Cape Town 7505, South Africa
[2] Department of Life and Consumer Sciences, College of Agriculture and Environmental Sciences, University of South Africa, Florida Campus, Johannesburg 1709, South Africa
[3] Navrongo Health Research Centre, Ghana Health Service, Navrongo P.O. Box 114, Ghana
[4] Faculty of Health Sciences, University of Limpopo, Polokwane 0700, South Africa
* Correspondence: perpetua.modjadji@mrc.ac.za

Supplementary Table S1. Search strategy on PubMed/MEDLINE.

Term number	MeSH terms	Number of records
1	Pregnancy Outcome	84,756
2	Pregnancy complications, infectious	49,766
3	pregnancy complication	470,460
4	pregnancy characteristics	0
5	pregnancy problem	0
6	HIV	106,895
7	HIV infections	312,850
8	HIV-1	84,262
9	HIV-2	4,322
10	AIDS	78,302
11	Acquired Immunodeficiency Syndrome	78,302
12	Anti-HIV Agents	59,275
13	HIV Protease Inhibitors	10,394
14	HIV Integrase Inhibitors	2,298
15	ARV	0
16	highly active antiretroviral therapy	22,536
17	ART	38,041
18	combination-ARV	0
19	combination-ART	0
20 (1,2,3,4, and 5)	((((((Pregnancy Outcome[MeSH Terms]) OR (pregnancy complications, infectious[MeSH Terms])) OR (pregnancy complication[MeSH Terms])) OR (pregnancy characteristics[MeSH Terms])) OR (pregnancy characteristics[MeSH Terms])) OR (pregnancy problem[MeSH Terms])) OR (pregnancy problem[MeSH Terms])	492,919
21 (6,7,8,9,10 and 11)	(((((HIV[MeSH Terms]) OR (HIV infections[MeSH Terms])) OR (HIV-1[MeSH Terms])) OR (HIV-2[MeSH Terms])) OR (AIDS[MeSH Terms])) OR (Acquired	347,641

	Immunodeficiency Syndrome[MeSH Terms])	
22 (12,13,14,15,16,17,18 and 19)	((((((((Anti-HIV Agents[MeSH Terms]) OR (HIV Protease Inhibitors[MeSH Terms])) OR (HIV Integrase Inhibitors[MeSH Terms])) OR (ARV[MeSH Terms])) OR (ARV[MeSH Terms])) OR (highly active antiretroviral therapy[MeSH Terms])) OR (ART[MeSH Terms])) OR (combination-ARV[MeSH Terms])) OR (combination-ARV[MeSH Terms])) OR (combination-ART[MeSH Terms]	111,510
23 (20, 21 and 22)	((((((((Pregnancy Outcome[MeSH Terms]) OR (pregnancy complications, infectious[MeSH Terms])) OR (pregnancy complication[MeSH Terms])) OR (pregnancy characteristics[MeSH Terms])) OR (pregnancy characteristics[MeSH Terms])) OR (pregnancy problem[MeSH Terms])) OR (pregnancy problem[MeSH Terms])) AND ((((((HIV[MeSH Terms]) OR (HIV infections[MeSH Terms])) OR (HIV-1[MeSH Terms])) OR (HIV-2[MeSH Terms])) OR (AIDS[MeSH Terms])) OR (Acquired Immunodeficiency Syndrome[MeSH Terms]))) AND ((((((((((Anti-HIV Agents[MeSH Terms]) OR (HIV Protease Inhibitors[MeSH Terms])) OR (HIV Integrase Inhibitors[MeSH Terms]))	174

	OR (ARV[MeSH Terms])) OR (ARV[MeSH Terms])) OR (highly active antiretroviral therapy[MeSH Terms])) OR (ART[MeSH Terms])) OR (combination-ARV[MeSH Terms])) OR (combination-ARV[MeSH Terms])) OR (combination-ART[MeSH Terms])) Filters: Free Full text, Clinical Trial, Randomized Controlled Trial, English	

Supplementary Table S2. Search strategy on Scopus.

**Search**	**Records**
(TITLE-ABS-KEY (pregnancy AND outcomes) OR TITLE-ABS-KEY (pregnancy AND complications) AND TITLE-ABS-KEY (HIV) OR TITLE-ABS-KEY (aids) AND TITLE-ABS-KEY (art) AND TITLE-ABS-KEY (ARV) AND (LIMIT-TO (DOCTYPE , "ar")) AND (LIMIT-TO (LANGUAGE , "English"))	**58**
